# A Case of Drug-Induced Liver Injury Associated With Saw Palmetto

**DOI:** 10.7759/cureus.115418

**Published:** 2026-08-29

**Authors:** Rakendu Jayasree Rajendran, Veesha Biscuitwala, Abhinav A Vayalveettil, Jashnaveer Singh Johal

**Affiliations:** 1 Internal Medicine, Virtua Health, Camden, USA; 2 Internal Medicine, Cleveland Clinic, Cleveland, USA

**Keywords:** benign prostatic hyperplasia, cholestatic hepatitis, drug-induced liver injury, hepatotoxicity, herbal supplements, saw palmetto

## Abstract

Saw palmetto (SP) is a widely used herbal supplement for benign prostatic hyperplasia (BPH) and is generally considered safe. However, rare cases of SP-associated drug-induced liver injury (DILI) have been reported. We describe a case of cholestatic hepatitis attributed to SP, highlighting the diagnostic challenges associated with herbal supplement use and polypharmacy.

A 70-year-old male with a history of BPH, hypertension, chronic obstructive pulmonary disease, atrial fibrillation, and cholecystectomy presented with one month of jaundice, pruritus, and pale stools. Physical examination demonstrated scleral icterus without abdominal tenderness. Laboratory evaluation revealed an alkaline phosphatase level of 215 U/L, total bilirubin of 7.3 mg/dL (direct bilirubin = 5.0 mg/dL), alanine aminotransferase of 305 U/L, aspartate aminotransferase of 133 U/L, and an international normalized ratio of 3.8 while receiving warfarin therapy for atrial fibrillation. Viral hepatitis serologies were negative. Right upper quadrant ultrasonography demonstrated mild biliary ductal prominence, and magnetic resonance cholangiopancreatography excluded biliary obstruction. Liver injury was initially attributed to congestive hepatopathy because of newly reduced left ventricular ejection fraction (34%-40%). However, the patient remained clinically euvolemic, liver biochemistries failed to improve, and progressive hyperbilirubinemia prompted further evaluation. A detailed medication history revealed over-the-counter SP use for approximately four months, discontinued two months before presentation, and a recent one-week course of trimethoprim-sulfamethoxazole for a urinary tract infection. Transjugular liver biopsy with measurement of the hepatic venous pressure gradient (6 mmHg) demonstrated cholestatic hepatitis with portal and lobular inflammation, eosinophilic infiltration, and bile duct proliferation, consistent with DILI. Right and left heart catheterization demonstrated normal filling pressures and the absence of obstructive coronary artery disease, making congestive hepatopathy unlikely. Warfarin was reversed with vitamin K and transitioned to apixaban because of concern for potential hepatotoxicity. The patient was treated with ursodiol and cholestyramine, resulting in gradual improvement in liver enzymes and bilirubin, and was discharged with close hepatology and cardiology follow-up.

This case highlights SP as a rare but important suspected cause of cholestatic DILI. Although causality cannot be definitively established because of concomitant exposure to other drugs, SP was considered the most likely implicated agent based on the temporal relationship with exposure, exclusion of alternative etiologies, compatible histopathologic findings, and clinical improvement after its discontinuation. Clinicians should routinely inquire about over-the-counter and herbal supplement use when evaluating unexplained liver injury. Increased awareness, comprehensive medication reconciliation, and improved regulation of dietary supplements may facilitate earlier recognition and reduce preventable hepatotoxicity.

## Introduction

Saw palmetto (SP; *Serenoa repens*), an extract of the dwarf palm native to the southeastern United States, is among the most widely consumed herbal products worldwide and is used predominantly for the symptomatic treatment of benign prostatic hyperplasia (BPH). Its proposed benefit in BPH is attributed principally to inhibition of type 1 and type 2 5-alpha-reductase, together with anti-inflammatory and antiandrogenic activity [1-6]. Despite its popularity, its clinical benefit remains uncertain: some trials and meta-analyses suggest modest symptomatic improvement, whereas Cochrane reviews found no meaningful benefit over placebo. SP is generally considered well tolerated, with gastrointestinal symptoms reported most frequently. Nevertheless, rare cases of liver damage and pancreatitis have been described anecdotally, including cholestatic patterns of injury. Published reports remain limited, and the diagnosis may be particularly challenging in patients with competing causes of liver-test abnormalities or exposure to multiple potentially hepatotoxic medications [7,8].

We report a case of SP-associated cholestatic drug-induced liver injury (DILI) in a patient using the supplement for BPH. The case adds to the limited literature by illustrating a clinically significant cholestatic presentation in the setting of cardiomyopathy and polypharmacy, where alternative etiologies required careful exclusion and structured causality assessment. It underscores the importance of specifically eliciting herbal and dietary supplement use when evaluating otherwise unexplained liver injury.

## Case presentation

A 70-year-old male with a past medical history of BPH, hypertension, chronic obstructive pulmonary disease (COPD), remote cholecystectomy, and recently diagnosed atrial fibrillation (AF) presented to the emergency department in November 2023 with progressive jaundice. He first noticed jaundice approximately one month before presentation, near the end of a cruise to the Bahamas. He denied abdominal pain but reported pruritus and pale stools over the preceding weeks. After jaundice had begun, he completed a one-week course of trimethoprim-sulfamethoxazole (TMP-SMX) for a urinary tract infection. He had recently started warfarin for AF.

A detailed medication history identified sequential exposure to two over-the-counter saw palmetto products used for BPH. From July through October 2023, the patient took Havasu Nutrition Saw Palmetto, one capsule daily with a meal, as directed on the product label. Each capsule contained a 500-mg saw palmetto blend comprising saw palmetto fruit powder and saw palmetto fruit extract standardized to 45% fatty acids; other ingredients were hypromellose and vegetable magnesium stearate. He subsequently switched to NOW Foods Saw Palmetto Berry Extract, once daily for approximately one to two weeks. Each softgel contained 160 mg of *Serenoa repens* berry extract; listed nonactive ingredients were bovine gelatin, water, glycerin, organic extra-virgin olive oil, and caramel color. It additionally contained stinging nettle root and pumpkin seed oil. He discontinued SP approximately two months before presentation. The chronology is described in Figure 1. No other herbal or dietary supplement use was reported. The patient also reported an unintentional 20-pound weight loss over the preceding six months. He consumed approximately nine alcoholic drinks per week, had quit smoking seven years earlier, and denied illicit drug use.

**Figure 1:**
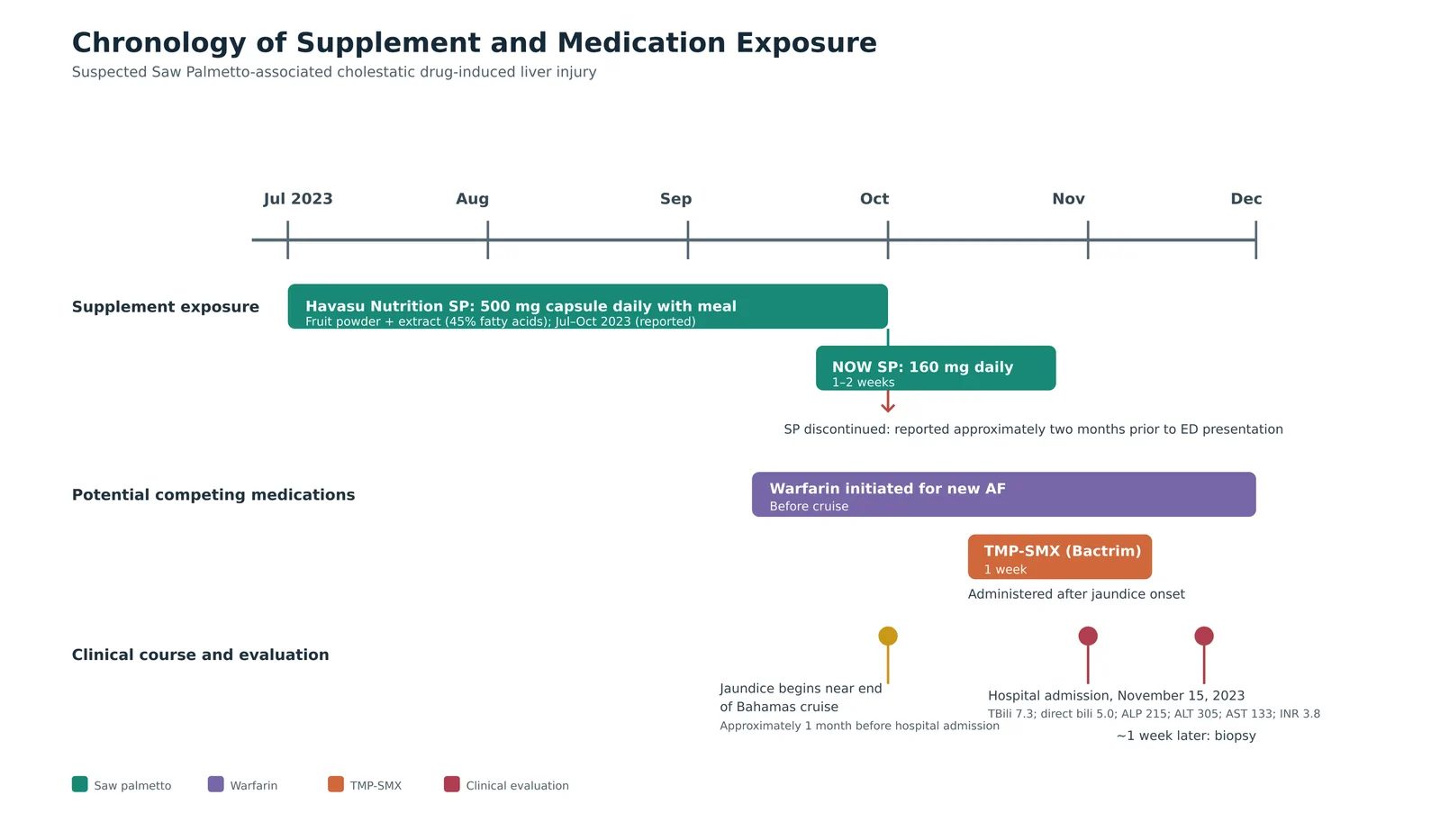
Chronology of saw palmetto and potential competing medication exposures relative to the onset and evaluation of cholestatic drug-induced liver injury. AF: atrial fibrillation; TMP-SMX: trimethoprim-sulfamethoxazole; TBili: total bilirubin; direct bili: direct bilirubin; ALP: alkaline phosphatase; ALT: alanine aminotransferase; AST: aspartate aminotransferase; INR: international normalized ratio.

On presentation, the patient was hemodynamically stable. Physical examination demonstrated scleral icterus without abdominal tenderness or other significant findings. Initial laboratory investigations are summarized in Table 1. These demonstrated a cholestatic pattern of liver injury with an alkaline phosphatase level of 215 U/L (reference range: 44-147 U/L), total bilirubin of 7.3 mg/dL (reference range: 0.2-1.2 mg/dL), direct bilirubin of 5.0 mg/dL (reference range: 0.0-0.3 mg/dL), alanine aminotransferase (ALT) of 305 U/L (reference range: 7-56 U/L), aspartate aminotransferase (AST) of 133 U/L (reference range: 10-40 U/L), and an international normalized ratio (INR) of 3.8 (reference range: 0.8-1.1) while receiving warfarin therapy. The calculated R ratio was approximately 3.9, consistent with a mixed liver-injury pattern with marked cholestatic features. Right upper quadrant ultrasonography demonstrated mild intrahepatic biliary ductal prominence within the left hepatic lobe and possible pneumobilia, findings considered compatible with his prior cholecystectomy. Serologic evaluation for hepatitis A immunoglobulin M, hepatitis B surface antigen, hepatitis B core immunoglobulin M, and hepatitis C antibody was negative.

**Table 1:** Initial laboratory investigations at presentation. Baseline laboratory findings obtained on admission demonstrated a cholestatic pattern of liver injury, elevated liver enzymes, hyperbilirubinemia, supratherapeutic international normalized ratio (INR), and negative serologic evaluation for viral and autoimmune liver disease. Bold values indicate abnormal laboratory results.

Laboratory test	Patient value	Reference range
Alkaline phosphatase (ALP)	215 U/L	44-147 U/L
Total bilirubin	7.3 mg/dL	0.2-1.2 mg/dL
Direct bilirubin	5.0 mg/dL	0.0-0.3 mg/dL
Aspartate aminotransferase (AST)	133 U/L	10-40 U/L
Alanine aminotransferase (ALT)	305 U/L	7-56 U/L
International normalized ratio (INR)	3.8	0.8-1.1
Hepatitis A IgM	Negative	Negative
Hepatitis B surface antigen	Negative	Negative
Hepatitis B core IgM	Negative	Negative
Hepatitis C antibody	Negative	Negative
Phosphatidylethanol (PEth)	Negative	Negative
Antimitochondrial antibody (AMA)	Negative	Negative

Warfarin was withheld because of the supratherapeutic INR, and vitamin K was administered. Hepatology was consulted for evaluation of jaundice and elevated liver enzymes. Magnetic resonance cholangiopancreatography demonstrated no biliary ductal dilatation or choledocholithiasis, with only incidental simple hepatic and renal cysts identified. Additional investigations showed a negative phosphatidylethanol (PEth) test, which made substantial recent alcohol exposure less likely. This, along with the history, makes alcohol as an etiology less likely. Antimitochondrial antibody testing was also negative, effectively excluding primary biliary cholangitis. The patient was started on loratadine and cholestyramine for symptomatic treatment of pruritus, with subsequent clinical improvement. Because of the recent diagnosis of AF, cardiology was consulted. Transthoracic echocardiography demonstrated a moderately reduced left ventricular ejection fraction of 34%-40%, moderate global hypokinesis, severe left atrial enlargement, mild pulmonary hypertension, mild mitral regurgitation, and trace tricuspid regurgitation. Electrocardiography demonstrated AF with rapid ventricular response and nonspecific ST-segment abnormalities. Given the newly identified cardiomyopathy, right and left heart catheterization was performed to evaluate intracardiac filling pressures and exclude obstructive coronary artery disease. The patient received aspirin and was transitioned to intravenous heparin before the procedure. Catheterization demonstrated normal right- and left-sided filling pressures with reduced cardiac output and cardiac index and no angiographic evidence of obstructive coronary artery disease. The normal right-sided filling pressure made congestive hepatopathy an unlikely explanation for the liver injury.

Following catheterization, the patient was transitioned to oral bumetanide and continued on metoprolol succinate 100 mg daily for ventricular rate control. After reversal of warfarin with vitamin K, his INR improved to 1.2, and long-term anticoagulation was changed to apixaban because of concern for potential warfarin-associated hepatotoxicity. He was discharged with close outpatient cardiology and hepatology follow-up. Follow-up laboratory investigations one week after admission are summarized in Table 2. Liver biochemistries remained abnormal, with an AST level of 70 U/L, ALT of 171 U/L, total bilirubin of 16.0 mg/dL, and direct bilirubin of 9.6 mg/dL. Because of persistent hyperbilirubinemia despite exclusion of biliary obstruction and improvement in cardiac status, concern for DILI increased. Hepatology therefore recommended transjugular liver biopsy.

**Table 2:** Follow-up laboratory investigations during hospitalization. Follow-up laboratory values demonstrating the trend in liver biochemistries and coagulation parameters during hospitalization following discontinuation of suspected offending agents and initiation of supportive medical therapy.

Laboratory test	Admission	One week later	Reference range
Aspartate aminotransferase (AST)	133 U/L	70 U/L	10-40 U/L
Alanine aminotransferase (ALT)	305 U/L	171 U/L	7-56 U/L
Total bilirubin	7.3 mg/dL	16.0 mg/dL	0.2-1.2 mg/dL
Direct bilirubin	5.0 mg/dL	9.6 mg/dL	0.0-0.3 mg/dL
International normalized ratio (INR)	3.8	1.2	0.8-1.1

Transjugular liver biopsy was successfully performed, obtaining three 20-gauge core specimens. Histopathologic examination demonstrated mild portal inflammation composed predominantly of lymphocytes with scattered eosinophils, lobular inflammation containing neutrophils, lymphocytes, and eosinophils, bile duct proliferation, and canalicular cholestasis without significant interface hepatitis or bile duct injury. The hepatic venous pressure gradient measured 6 mmHg, indicating the absence of clinically significant portal hypertension. Collectively, the histopathologic findings were most consistent with cholestatic DILI, with SP considered the most likely causative agent based on the temporal relationship between exposure, exclusion of competing etiologies, and clinicopathologic correlation. However, warfarin as a cause cannot be excluded, although unlikely, as warfarin as a single agent causing cholestatic DILI is exceptionally rare. Supportive therapy with ursodiol and cholestyramine resulted in gradual improvement, with laboratory values closer to discharge detailed in Table 2. Notably, bilirubin, though improved, continued to remain elevated at discharge.

## Discussion

This case illustrates the diagnostic and management challenges of DILI in a patient with a complex medical history, polypharmacy, and herbal supplement use. Our patient's presentation with jaundice, pruritus, and pale stools in the absence of significant abdominal pain was consistent with a cholestatic pattern of liver injury, highlighting the heterogeneous clinical manifestations of DILI. SP is a widely used herbal supplement marketed for the treatment of BPH. Although generally regarded as safe, it is not approved by the U.S. Food and Drug Administration (FDA) for the treatment of BPH because of insufficient evidence supporting its efficacy [9]. Early clinical studies suggested that SP might improve lower urinary tract symptoms; however, subsequent systematic reviews and meta-analyses have questioned these findings [10,11]. In particular, a comprehensive meta-analysis published in 2021 that included 27 randomized controlled trials demonstrated with high certainty that SP provides little to no clinically meaningful improvement in urinary symptoms or quality of life in either the short or long term [12].

Serious adverse events associated with SP are uncommon. However, hypertension has been reported in approximately 3.1% of participants in one study [13]. Additional reports have described acute pancreatitis, intraoperative bleeding, and, rarely, hepatotoxicity associated with its use [14-20]. Although uncommon, these reports emphasize that herbal supplements should not be assumed to be inherently safe. DILI remains a diagnosis of exclusion, requiring careful evaluation to eliminate competing causes of liver injury. In our patient, viral hepatitis, autoimmune liver disease, alcohol-related liver disease, biliary obstruction, and congestive hepatopathy were systematically excluded. Viral hepatitis serologies, phosphatidylethanol (PEth), and antimitochondrial antibody testing were all negative, while magnetic resonance cholangiopancreatography demonstrated no evidence of biliary obstruction. Although the patient was found to have newly diagnosed cardiomyopathy, right and left heart catheterization demonstrated normal right- and left-sided filling pressures and no obstructive coronary artery disease, making congestive hepatopathy an unlikely explanation for his hepatic dysfunction.

Establishing the causative agent was particularly challenging because the patient had been exposed to multiple medications with potential hepatotoxicity, including warfarin and TMP-SMX, in addition to SP. Nevertheless, the temporal relationship between SP exposure and symptom onset, exclusion of alternative etiologies, histopathologic findings, and subsequent clinical improvement following discontinuation collectively supported SP as the most likely causative agent. Though stinging nettle root and pumpkin seed were constituents of the NOW brand SP formulation that the patient took, they cannot be fully excluded but are unlikely to cause DILI in this patient, given that there have not been any reported cases of the same. This case highlights the importance of obtaining a comprehensive medication history that includes prescription medications, over-the-counter products, and herbal supplements, as these agents are frequently overlooked despite their potential to cause clinically significant liver injury. The decision to perform a liver biopsy proved essential in establishing the diagnosis. Histopathologic examination demonstrated mild portal inflammation composed predominantly of lymphocytes with scattered eosinophils, lobular inflammation, bile duct proliferation, and canalicular cholestasis, findings characteristic of DILI. The absence of significant interface hepatitis or bile duct injury further helped exclude alternative causes of liver injury and strengthened the clinicopathologic diagnosis. The cornerstone of DILI management is prompt identification and withdrawal of the offending agent. In our patient, discontinuation of SP was followed by gradual improvement in liver biochemistries, further supporting its role as the causative agent. Supportive therapy also played an important role in symptom management. Ursodiol was administered for its potential choleretic effects, while cholestyramine provided symptomatic relief of pruritus. Warfarin was discontinued and replaced with apixaban because of concern for potential drug-related hepatotoxicity and the patient's supratherapeutic INR.

The increasing use of herbal and dietary supplements has been accompanied by a growing number of reports describing possible supplement-associated hepatotoxicity. This trend may be concerning, given the limited regulatory oversight of many over-the-counter products. Furthermore, the increasing incidence of hepatotoxicity associated with Ayurvedic and other alternative medicines has become an important public health concern [20]. These observations underscore the need for clinicians to maintain a high index of suspicion for herbal supplement-related liver injury and to routinely obtain a comprehensive medication history that includes complementary and alternative therapies. Greater clinician awareness, improved patient education, and continued efforts to strengthen the evaluation and post-marketing surveillance of dietary supplements are essential to reduce preventable cases of hepatotoxicity and improve patient safety.

## Conclusions

Our case reinforces the necessity for clinicians to maintain a high index of suspicion for supplement-related hepatotoxicity and underscores the importance of a comprehensive evaluation of all medications and supplements in patients presenting with liver dysfunction. It highlights saw palmetto as a rare but important suspected cause of possible cholestatic DILI. Although causality could not be definitively established because of concomitant exposure to other drugs, saw palmetto was considered the leading suspected agent based on the exposure history, exclusion of alternative etiologies, and improvement after stopping, though warfarin as a cause cannot be fully excluded. The case also illustrates the diagnostic challenges posed by polypharmacy and variable supplement formulations. Clinicians may need to routinely obtain a detailed, product-specific history, including formulation, dose, duration, and timing, when evaluating unexplained liver injury. Greater awareness, comprehensive medication reconciliation, clearer product labeling, and patient education may facilitate earlier recognition of supplement-related toxicity and reduce preventable harm.
